# Dental Organizations

**Published:** 1891-08

**Authors:** Walter Harrison

**Affiliations:** Brighton, England


### DENTAL ORGANIZATIONS.[1]

BY WALTER HARRISON, L.D.S., D.M.D., BRIGHTON, ENGLAND.

I HAVE often thought that the large number of dental societies in the United States of America would lead to better results and saving of energy if they were branches of a national association, as the American Dental Association. Not being with you, I do not wish to draw comparisons, but I should like to give a brief outline of our leading organizations in England.

In all instances the teaching is conducted at a hospital, either a special institution or the dental department of a general hospital; that is, the general public recognize the necessity of dental operations in the poorer sections of the community, and liberally support these charitable establishments by means of voluntary subscriptions, donations, or legacies. Dental hospitals are now fully recognized as one of the charitable institutions of the country. The point I wish to emphasize is *the public interest.* The Committee of Management is elected from and by the subscribers, and the public financial support is relied upon more than fees from pupils (there exist dental hospitals and departments which are conducted by the honorary dentists only, no student being admitted). In return for the subscriptions the benefactor is entitled to a certain number of "letters of recommendation," to be presented by the applicants for special operations,—viz., fillings, anæsthetic operations, regulation cases, etc.; ordinary extractions, and cases of accidents, fractures, etc., are admitted without such introduction.

The minor details of the working are similar to those with which you are familiar. There exists the hospital and school, engaging really in same work and building, and co-operating with perfect harmony. Nearly all offices are honorary and are eagerly sought after by young practitioners, and are looked upon as positions of distinction.

The dental surgeon appointed to general and special (not dental) hospitals occupies the same position as the physician and surgeon. Nearly every medical charity in the United Kingdom has upon the staff an honorary dentist.

After the Dental Bill passed and became law, the "Reform" Committee resolved itself into the British Dental Association.

---

[1] Read before Harvard Odontological Society, November 29, 1890, by James Shepherd, D.M.D., Boston, Mass.

EXTRACTS FROM MEMORANDUM OF ASSOCIATION AND BY-LAWS.

The objects for which the Association is established are the promotion of dental and the allied sciences, and the maintenance of the honor and the interests of the dental profession by

"The periodical meetings of the members of the Association and the dental profession generally in different parts of the country.

"The publication of a periodical journal, and by

"The maintenance of the spirit and provisions of the Dentists' Act, by such lawful means as may be necessary, etc."

EXTRACT FROM THE BY-LAWS.

"A person who is registered in the Dentists' Register shall be eligible for election as a member of the Association, provided that he be of good character; that he does not conduct his practice by means of the exhibition of dental specimens, appliances, or apparatus in an open shop, or in a window, or in a show-case exposed to public inspection; or by means of public advertisements or circulars, describing modes of practice, or patented or secret processes; or by the publication of his scale of professional charges.

"Any registered practitioner not disqualified by any by-law, who shall be recommended as eligible by any three members of the Association (the recommendation of one being from personal knowledge), and who has signed the appended form of application for admission and agreement as to terms of membership, may be elected a member by the Representative Board or by a committee appointed for that purpose by the board, or by the council of a recognized branch."

The subscription is one guinea per annum, and each member is entitled to a copy of the journal of the Association monthly, and to attend the annual meetings of the Association.

The Association is the real representative of the profession, and is divided into eight (at present) branches, each having a president, secretary, and council (for all practical purposes they are societies in themselves).

The meetings of the various branches consist of the annual meeting for election of officers, etc., and to fix place of next gathering, in addition to the regular programme usual at such societies. Minor meetings are also held according to the demands of the district.

The Association also possesses a Benevolent Fund to aid distressed dentists' widows and orphans.

The Representative Board consists of all the presidents and secretaries of the branches and a certain number of members elected by ballot, who are the body of the Association.

One of the most important features is the possession of the Dental Journal; thus for about $5.25 annually a member is really a shareholder in the publication.

It must not be supposed that the British Dental Association is the only society we possess, several of the larger towns having one, and are able to maintain excellent scientific meetings, but at the same time the British Dental Association is represented everywhere.

Personally I should like to see the American and Southern Dental Associations become one body (*vide* INTERNATIONAL DENTAL JOURNAL, September, 1890), and every State society become a branch of the American Dental Association, publishing a journal of its own.

The degrees are granted by the various colleges of surgeons, and their diplomas are recognized by the state, so that the possessor of the dental license (L.D.S.), which the law *demands*, entitles the holder to be registered upon payment of the fee without further and (I consider) unnecessary examination. In my mind the diploma of any of the recognized dental schools should entitle the graduate to practise in any State. If State examiners were to be present at the college examination the difficulty would be overcome.

